# Miscellaneous

**Published:** 1880-01

**Authors:** 


### MISCELLANEOUS.

### OPENING NIGHT OF THE COLUMBIA VETERINARY COLLEGE.

A large and select audience was gathered in the lecture-room of the Columbia Veterinary College on October 1st, at 8 o'clock P. M., to witness the opening exercises. These were opened by Dr. Alexander Hadden, the President of the Faculty, by the following remarks:

In behalf of the Board of Trustees and Faculty of the Columbia Veterinary College, I take pleasure in announcing that the second regular session opens this evening auspiciously; that the Trustees, Faculty and Alumni are all in harmony and strictly loyal to the true interests of each other and of the College; that the institution, though young, is strong, healthy and active, and free from the encumbrance of debt: hence the struggle with its rivals for preemininence in the future will be easier and more certain. Those who know well our skilled teachers will not doubt the issue. For the purpose of clearness I will now state that the horse is not the only animal to be studied here, but with him every other creature, even man comparatively. I need not here mention the names of those who will conduct these studies nor introduce you to their personal attainments, for the future will do this more effectually than I can, and no doubt by enrolling their names on the scroll of honor with those of Huxley, Darwin, Tyndall, Youatt, Fleming, Wilson, Granger, and others of no less fame. In closing I will take the liberty of congratulating the College on the late acquisition of teachers. Such names as Satterthwaite, Porter, Berns and Heath cannot fail to give to the scientific and practical interests of the institution unprecedented advantages. With these few remarks, as introductory, I will introduce Prof. Hawkins, one of the Faculty, who will now deliver the opening address.

Then followed the

### OPENING LECTURE OF PROFESSOR THOMAS H. HAWKINS.

MR. PRESIDENT, *Gentlemeu of the Board of Trustees and Members of the Faculty. Ladies and Gentlemen :*

The selecting of a subject for the introductory address which is such a time-honored feature of the opening-night has been a matter of no little difficulty.

In the routine pursued on such occasions as the present, we ordinarily have a panegyric or a laudatory discourse on the life and rewards of the medical man; thus exciting and stimulating the medical student to ambitious aims.

There are many questions of practical importance and interest to the student, such as the best disposition of time, the proper methods of study and habits of life. But there are many of my colleagues better fitted to discourse on matters of this kind, and they will doubtless, during their course of lectures this winter, enlighten you upon these subjects, giving you all the necessary information and guidance.

But there are other questions, which inasmuch as they are of equal interest to the laity as to the veterinary profession, may furnish a fit subject for an address to a mixed audience.

Among these there is one regarding which the scientific Veterinarian is properly called on to enlighten the public; to prevent on the one hand the abuse of man's mastery of the brute creation; on the other as strongly to oppose the morbid and high-strung commonplaces of pseudo-philanthropists.

Only a few days since there appeared in the *N. Y. Herald* (Sept. 21st.) an article titled, "*Science Indeed,*" addressed to his honor, Mayor Cooper, emanating from the pen of one of our sensationally sympathetic philanthropists, in which were depicted horrors and tortures imaginary, that have been, and were to be, perpetrated on the unfortunate canines by the "Chief Surgeon of Bellevue Hospital." The author of this article evidently knows nothing of the workings of hospitals or colleges. The surgeons of Bellevue Hospital have not practiced vivisection, so far as I know, nor do I believe there *ever* was a vivisection practiced on the dog in Bellevue *Hospital.* The Physiologist of Bellevue Hospital Medical College is not connected with the Hospital.

This writer says "why not take measures to stop these atrocious tortures, as they have done in other countries," and then the only case which he cites from another country is one in which a man tortured a dog, punched his eyes out, plugged his ears, and wondered why the dog took no notice of him. Whether this was a case of vivisection or not, I leave for you, gentlemen, to decide.

This writer further claims to be a great admirer of the brute creation, yet the severest epithet he could apply to these experimentators is *to call them brutes.*

Vivisection is referred to by the same writer as an ancient and barbarous practice. It is probably unnecessary for me to tell you that vivisection was not practiced to any very great extent until the latter part of the last and during the present century. A few vivisections were performed, it is true, prior to this time, but our medical literature has grown rich in facts only through the numerous and varied vivisectional experiments of *strictiy modern* physiologists. The great mother ideas of medicine, now at the command of every well-informed and skilled physician and surgeon, were absolutely unknown to the ancients.

The simple experiments by Galen and a few others, through a long lapse of time, finally culminated in that grand discovery by Harvey, in 1616; thus these simple, though somewhat vague experiments, step by step, led to important discoveries, awakening the physicians and surgeons of the time to the consciousness of the fact that the only way to ascertain the normal working of living organisms was to study their phenomena on living bodies, and this could only be done by vivisection. The sentimentalists are most painfully and deplorably ignorant of the methods employed in performing vivisections, and of the knowledge obtained, either directly or indirectly, from experimental physiology, or they would not have compared the pulling of a tooth from the wheel of a chronometer to the stretching of a dog's nerve with a pair of pincers. There are a number of exaggerated and ridiculous statements in the article referred to, but I defer further mention of them for the present.

If we had only scientific and well informed men and women to deal with, it would be absurd to take *any* notice of the appeal to the Mayor, but the great mass of the people are not informed on this subject, and know but little about physiology, and still less about experimental physiology. The public sentiment has become biased and prejudiced against those who experiment on living animals, and this state of affairs has been brought about by articles eminating from enthusiasts, riders of hobbies, oftentimes narrow-minded men who are over sensitive and morbid, not unfrequently suffering from a monomania.

Taking these things into consideration, it becomes our duty as disciples of comparative medicine to inquire into this subject in a methodical and systematic way.

First let us see by comparison what the physician of to-day would know, minus the knowledge he has attained, either directly or indirectly, from vivisection,

What did we know of the phenomena of digestion before Bassow's (1842) and Blondlot's (1843) experiments on dogs by means of gastric fistulae? Absolutely nothing, except a few facts made known by Dr. Beaumont's observations on the unfortunate Alexis St. Martin in 1825. Imagine a physician treating indigestion without the slightest knowledge of the phenomena of *normal* digestion; and yet that would be our condition to-day if the antivivisectionist fifty years ago could have had his way and suppressed vivisection.

In 1616 Harvey, by experimenting on *living* animals, discovered the circulation of the blood.

In 1661 Malpighi, experimenting on the lungs of a *living* frog, and a little later Lieuwenhoek, on the wing of a *living* bat, demonstrated the capilary circulation. Thus the grand discovery of Harvey was begun and completed by the aid of vivisection.

Alexander Walker might have made the greatest discovery since that of Harvey had he believed the result of his experiment upon a *living* animal in 1809. Walker stated that his experiment showed that the posterior roots of the spinal nerves were for sensation, and the anterior for motion, but by a process of reasoning he was convinced that the posterior were for motion, and the anterior for sensation. Walker's reasoning, independent of proof and demonstration, may have served him a very good purpose in theoretical deduction, but deprived him of the honor of one of the greatest discoveries in physiology.

Sir Charles Bell, in 1811, after some experiments on the spinal nerves, *not* on *living* animals, however, published a pamphlet for private circulation among his friends, in which he stated that the anterior roots were for both motion and sensation, and that the posterior presided over "the secret operations of the bodily frame, or the connections which unite the parts of the body into a system."

Walker, Bell, and all others, had in the true facts failed, it remained for Magendie to demonstrate to the world, in 1822, the difference in function between the posterior and anterior roots of the spinal nerves.

Bell failed. Why? Because being opposed to vivisection he experimented on *dead* animals. Magendie succeeded; he experimenting on conscious *living* animals

This horrible, barbarous, and *very* ancient experiment, as "animaltarians" term it, was really the birth of our knowledge of the physiology of the nervous system. None have contributed more in this direction than our illustrious friend, Chauveau, who began his experiments, in 1861, upon horses, dogs, and other inferior animals. I

may here remind you that *veterinarians* of the past have contributed much to our medical literature, and we may expect much more from them in the future; much, I trust, may come from the Alumni of the "Columbia Veterinary College."

From this short sketch of the history of the early discoveries in physiology, you can readily understand that we could know nothing of the physiology of the nervous system, of the circulation the blood, or of the digestive organs, without experimenting on *living* animals. But I have wandered somewhat from the question at issue. What experimental physiology has done for medical literature is not so much a question for us to consider, as it is, whether it is right, humane, desirable, or even advisable, that the physiologist should continue to practice what is generally termed vivisection.

What do we understand by vivisection? We may define vivisection to be experimenting on the still living, though not necessarily conscious animals with instruments, or by any other means that will enable the investigator to see or demonstrate in the best manner possible the phenomenon or phenomena sought for. It would be well for us in this connection to explain the term "living." The agitators against vivisection have not apparently a clear comprehension of the meaning of this word. They do not know that an animal may be dead, and yet alive for all purposes of the physiologist.

From the standpoint of the physiologist there are three chief stages in life. First, there comes a time when all conscious volition, sensation, and existence is lost; that is, there is existence, but not conscious existence; volition, but not conscious volition; possibly sensation (doubtful), but positively not conscious sensation. Second, a time when the respiratory efforts stop, and the heart ceases to act. Third, when the muscles all become rigid, but still retain some of their irritability. Somatic death is only complete when all irritability is lost; then follows molecular death, slowly, fortunately for the physiologist, in the cold-blooded animals. These events follow each other always in the same order, the time elapsing between any two of them may be short, or of considerable duration.

This question of vivisection, which is of so much importance, might be considered from many points of view. We might remind the antivivisectionist that all the pain ever wrought upon animals for the advancement of scientific knowledge is but a trifle when compared to the tortures inflicted by the sportsman in the pursuit of his game or sport. We might remind this gentle, tender-hearted, and noble sport or huntsman of the horrors and tortures so often witnessed by him in the field and forest; yes, even in the pigeon-match, and ask him if he ever followed one of those poor wounded birds, and saw it endeavoring to hide its maimed and crippled body. We might picture to him the lingering death of this suffering, forlorn, and starving bird. Why, gentlemen, all the pain ever inflicted in the name of experimental physiology is less than that brought about by the hunter or sportsman within a twelfth month.

But this style of argument, though based upon facts, does not justify vivisection, nor is it any use to argue thus—except to stop the mouth of the sportsman—with those who oppose vivisection, because their opposition and the ultimate doing away with vivisection, if possible, is but the stepping-stone with them to *the* great anticipated move against sport itself.

The question of the right and wrong of vivisection has been advanced by calling attention to these facts. Still the question remains, *is it right* for us to inflict pain, either *with* or *without death*, on the inferior animals for the advancement of useful knowledge.

Before going further in our argument we might just here remind the eaters of meat that the pain caused in one slaughtor-house of this city within the last week is greater than all the pain ever wrought upon animals by the combined army of the physiologists of the past.

Yet who dare to dispute the right to slay animals, to provide food for the nourishment of our bodies? Time forbids further discussion in this direction, and we must hasten to argue this question out on its own merits. Then first let us consider it from the aspect of the animal.

Our life is in reality a struggle for existence, and in this struggle we not only have to contend with plants and lifeless forces, but with the living inferior animals. If we are to succeed we must control and use them, thus the nature of things *forces* upon *us* the burden of government, for *we* must *rule* or succumb. We must use and kill animals, or animals will destroy us. Then, if by the condition of our existence, it becomes our *duty* to use and to slay animals, the question arises, how to use, and when to kill.

Certain great principles, *do* and *must* necessarily, govern us in our relations and conduct toward inferior animals. Three or these general principles I will mention for our present consideration, namely: It is *nccessary* to kill for *self-preservation*, that we may be properly fed and clothed; and for the advancement of knowledge, which could nct otherwise be obtained. These three principles are included in one broad principle, that is: The good to the human race should be the rule of the individual, which ordinarily is to govern him in his conduct toward inferior animals. In regard to the first statement, it will be well perhaps for us to inquire what is meant by self-preservation. Whenever the death of an animal, with or without pain, is beneficial to the human race, then the slaying of that animal becomes a duty, and we are justified in performing that duty.

It is *impracticable*, if not *impossible*, to draw a line, justifying the slaying of an animal for a particular purpose, and condemn it for another. In this selfish world; that is, *apparently* selfish, the guiding principle, not only of man, but alsò of the inferior animals, is the struggle for existence, self-preservation. But it evidently was intended for man to rule and govern the world. Were it proper to drag sacred things into a discourse of this kind, I could quote extensively from the Bible to substantiate this view. Prevent the slaying of animals by man and what would be the result. Imagine for a moment what would happen if the entire human family were organized into a society for the prevention of the destruction of the canine tribe. What a grand army of howling and ferocious dogs we would have within a few generations, who would either destroy man, or compel him to take up arms in self-defence.

I fear the dogs would be sufficient in number to vivisect the entire human race. Picture to yourself the brave, patriotic president of this society surrendering his body a living sacrifice to the cause which he had so nobly defended. Suppose this society to go still further and enact a law prohibiting the destruction of any kind of game in the United States, say for the period of thirty years, you can readily understand the farmers' doom; or again, suppose they prevent the destruction of insects and vermin which infest houses and beds. Possibly some of you know only too well what your fate has been when compelled to accupy temporarily places thus infested. Self-preservation is one of the first laws of Nature. Then, since man must rule or be destroyed, our opponents must concede to us this first general principle, thereby granting man the *right* to slay animals.

The second general principle which we have laid down is conceded by all. Then if it is necessary that we should use and slay animals that man may be properly fed and clothed, it is equally necessary that he experiment on living animals, in order to obtain an accurate knowledge of life, since much of his success in this strife for existence depends on a thorough knowledge of the phenomena of organized bodies, and inasmuch as we have shown that this knowledge cannot be obtained without the inflicting of death and pain, man is therefore justified in vivisection.

Whenever we cause death or pain on animals which does not result in benefit to man, then and then only are we guilty of cruelty. It seems to me desirable that we, who are seeking to advance knowledge, who are teachers and students of comparative science, with the avowed purpose of preventing and curing disease, thereby relieving and preventing much pain and suffering, should have a clear understanding of what our conduct is to be toward animals, for this reason I have gone rather minutely into detail, and also with a view to refute the absurd statements brought forward by those who are ignorant in matters pertaining to this subject.

During this lecture, whenever I have had occasion to refer to the death caused by vivisection, I have associated the word pain for the reason that the public have an idea that vivisection means cruelty and pain, not death at all, whereas, in reality, in the great majority of cases, death only is caused, there being no pain whatever. Much of this confusion has grown out of the erroneous conceptions of life, to this I alluded in the earlier part of the lecture. What a surprise, indeed, a revelation to Mr. Bergh, his followers, and others to learn that a skilled physiologist can keep a dog for hours, if necessary for the success of his experiment, with its heart in perfect action, respiration being carried on by and under the control of the experimenter himself, by means of a bellows, connected with the trachea, the animal entirely unconscious, not even required to do its own breathing.

It would be absurd to speak of pain in such an experiment as this, would it not? And yet operations performed on an animal in this condition are not only highly instructive, but by far the most frequently performed.

Physiologists of to-day use anæsthetics whenever it is possible, and when properly administered, pain is out of the question. Animals will howl, and sometimes struggle violently, but those who are familiar with the three stages in the administration of an anæsthetic know perfectly well that these are *not* indications of pain. Occasionally it is necessary to perform experiments, vivisectional in character, where it is not advisable to use anæsthetics; these are comparatively few in number, and may be divided into two classes. The first are usually short, the pain very slight, and are the more numerous.

In the second, there is considerable pain, quite severe in some cases, but these are becoming less numerous, and if the science of physiology is not turned aside by any false issue, the time will come whe nthe physiologist will produce pain only when *pain itself* is the object of study. After this discussion of the subject we conclude that the physiologist causes death, some *slight* pain and a little severe pain. Perhaps a very active physiologist might cause as much death and pain in an ordinary lifetime as a village butcher does in twelve months.

What justification do we plead for all this death and slight amount of pain? In reference to this question I have endeavored to show that the only knowledge which we possess of the laws of life was obtained through and by this death and pain. I attempted in my sketch of the history of experimental physiology to show that experiments on living animals were the stepping-stones of physiological progress.

It would be impossible in an address of this kind to give even a mere synopsis of *all* the knowledge obtained through vivisectional experiments. But let us glance for a moment at this immense treasure. To begin with, there are the discoveries of the immortal Majendie and Harvey, already alluded to; then there is the keen intellectual Haller, who established the doctrine of irritability; the accurate and skillful Claude Bernard, who demonstrated the glygocenic function of the liver, and unraveled the mysteries of the pancreas, and who also prepared the way for the discovery of the system of the vasor-motor nervse, completed by Brown-Sequard.

Our own renowned countryman, Austin Flint, Jr., who demonstrated the excretory function of the liver, thereby giving the first clear insight into the pathology of a disease now known as cholesteremia, besides his experiments on the elimination of urea; also, his ingenious researches demonstrating the respiratory center, which, while they are not conclusive, are interesting and will doubtless lead to a satisfactory solution of this much disputed question. Prevost and Dumas, who demonstrated the function of the kidney, showing that urea was not manufactured in the kidney *per se.* Longet, who demonstrated muscular irritability. Marshal Hall, who demonstrated the importance of reflex action, besides a host of others whose names I will merely mention, such as Helmholtz, Reiset, Du Bois-Reymond, Marey, Aeby, Edwards, Regnault, Max Pettenkofer, Belini, Bush, W. H. Mason, Liebermeister, Funke, Bowman, Weber, Pflüger, Flourens, Vulpian, Lavoisier, Dalton, Carpenter, Arnold and others.

Take away from physiology all this experimental knowledge, and what would we have left? There are many questions yet which must be solved in the physiological aboratory. Modern physiologists are now in a fair way, and will soon clear up all those mysteries connected with the words inflammation and fever.

In what way does physiology benefit mankind? This question, I think, has been fully answered, but a few words added in this connection will not be amiss. Most assuredly physiology is the basis for the conduct of life, but we are more interested in its relation to the practice of medicine.

Besides the vast amount of general knowledge for which the medical profession is indebted to physiology, there are many special discoveries, such as the now scientific methods of controling hemorrhage. The use and introduction of the ligature in the controling hemorrhage was brought about by experiments on dogs and other inferior animals, and its application in the cure of aneurism was made known by John Hunter, that eminent physiologist as well as the father of modern surgery.

Some of you have witnessed surgical operations, and you have seen times of danger during these operations, when it seemed as if the last drop of blood would gush forth from the divided or wounded arteries. But no; the calm and skilled surgeon seizes the artery, and the hemorrhage is permanently arrested by a properly applied ligature, by torsion or other means. Thanks to vivisection.

But how was it of old, when the hot searing irons, and other cruel, rude, barbarous means were resorted to? If the anti-vivisetionist had had his way such would be the state of things to-day. When for reason of great loss of blood, or deficiency of blood cells from other causes there is danger of immediate death, we can now resort to transfusion of blood; another one of the special discoveries of physiology.

We are also indebted to physiology for the modern and improved treatment of inflammation, as well as many other new ideas in pathology. I might go on enumerating other benefits without limit, but time will not permit.

We have yet to consider the influence of vivisection on man himself. Is it demoralizing or not? Does it render him hard-hearted and cruel? Experience teaches quite the reverse. Physiologists, who have experimented on *living* animals for the purpose of gaining new truths, have invariably become more gentle, and necessarily careful and tender. Physicians and surgeons, almost universally, are noted for their kindness and sympathy for their skill in delicate manipulations, which come, from experience. If they have had sufficient vivisectional experience they can cut within a fractional part of an inch of their dearest friend's life, if necessary to save that life. Wherever they can prevent suffering, or cruelty in the human family, or even among the inferior animals, they are ever ready to lend a helping aid, oftentimes sacrificing *their* lives for the good of others. Indeed, they live for humanity.

What can we say for our opponents—the anti-vivisectionists? They would do away with all the civilized methods of punishment for human beings, and substitute the whipping-post. Spend their time in developing their muscles, the triceps particularly, and in cultivating endurance, while their leader himself would engage in the inventing and constructing of a whipping machine, lest muscle and endurance fail when the time comes for applying the lash. If a human being offends, strikes a dog for instance, he would have him whipped into submission. Why? Because man has the power of reasoning. If the dog offends, he would have him reasoned into submission. Why? Because the dog *cannot* reason.

In the *Herald*, of September 30th, appeared another one of these overdrawn and sensational articles. Michael Servetus was a man after its writer's own heart, therefore it is not strange that he should endeavor to give him the credit of the discovery of the circulation of the blood. Servetus was regarded, even at that early date (1553), as a sensational and spiritualistic lunatic. Calvin finally caused Servetus to be burned alive, and his sensational books on theology committed to the flames with him.

It is rather painful to have to inform a gentleman, of the standing of the President of a Humanitarian Society, that *Columbus* discovered *America* and not the circulation of blood. It would be well perhaps for this man to postpone his article on pedestrianism, and brush up a little on United States history and primary geography.

A. Padua, Professor of Anatomy, Columbo and Cesalpinus, of Pisa, spoke of the passage of blood through the lungs, but this was not the discovery of the circulation of the blood. Shakespeare spoke of the tide of blood, condemned vivisection, and died in 1616. Says this writer: "Harvey practised vivisection and discovered and demonstrated the circulation in 1616, and not in 1628 as stated by Bergh.

After all this writer seems inclined to give Harvey the credit of this discovery, if he and vivisectionists generally will only admit that it was reasoned out. But no, guess-work will not answer the purpose of the physiologist, they want facts and facts only.

I here state most emphatically, and I am prepared to defend the statement when called upon, viz: that there never has been a physiological problem solved without the aid of vivisectonal knowledge from the time of Galen to the present moment.

The same writer suggests a moral, also demanding that vivisection be done away with, and the defenders of God's creatures see to it, that the doors of the State's prison be close upon all those who seek to torture or spill canine blood. We would suggest something quite different, in fact the time has come, in the period of the history of our country, when the public demand and are calling for educated and cultivated veterinarians. They and they only will be able to disabuse the minds of the people of the prejudices, false ideas, and superstitious notions, which have been inculcated by such men as are now presiding over the present Humanitarian Societies and other benevolent institutions. Let us instead of going to State prison see that every position of honor in the different societies, for the prevent of cruelty to animals, and other similar institutions, be filled by accomplished, skillful and learned veterinarians. Yes; let even the President be a high-toned, hororable, truthful veterinarian, well versed in all that pertains to comparative medicine and surgery, and better still, a skillful and humane vivisectionist. But where are these men to come from? Shall I say that the Columbia Veterinary College will furnish its full quoto? The earnest and fervent desire of your instructor in physiology and surgery is that she may, and that her stndents, take as high rank scientifically as any who have ever held the degree of "Doctor of Veterinary Science."

This address was enthusiastically applauded. Among the guests of the evening were remarked Dr. Stephen Smith, Dr. Satterthwaite, Dr. E. H. M. Sell, Dr. Vollum, U. S. Army, Mr. Conklin, Superintendent of the Central Park Zoological Department, and numerous other prominent physicians and other citizens of the city.

---

A NATIONAL BUREAU OF VETERINARY INSPECTION.—At the annual meeting of the American Veterinary Association, held recently in this city, a proposition to establish a National Bureau of Veterinary Inspection was made. It was proposed that the Bureau have similar powers as regards animals with those possessed by the National Board of Health. The disease, which it is thought, more especially calls for such an organization is pleuro-pneumonia. It is urged that recent events have shown how valuable it would be to large commercial interests to have a central bureau that might keep cattle dealers constantly informed as to the prevalence of contagious diseases. Such an arrangement would prevent panics, and render impossible the prohibition of American cattle by European Governments, upon merely sensational reports.

It is, on the one hand, said that such information can be secured without Government aid. Furthermore, the present status of veterinary medicine is so undefined, that a National Bureau would not have much more of a legitimate professional basis than a National Bureau of Barbers to keep themselves informed upon sycosis. There is in this country only one veterinary college which exists under legislative sanction, and which can grant genuine diplomas. Veterinary practitioners, therefore, are composed of three classes: persons who have graduated from regular foreign and the one regular home school; persons who have graduated from other home schools, and have received diplomas which are virtually only certificates; and third, persons having no regular education whatever.

It is feared that the establishment of a National Bureau out of these elements would produce endless quarrelling without securing any valuable results.—*New York Medical Record*, November 3th, 1879.

*An article* in the December number of POPULAR SCIENCE MONTHLY treats of the subject of many toed horses. It is accompanied by an illustration of a pertinent case—a horse with a well developed hoof on the inside of the usual hoof, and symmetrically on both the anterior and posterior extremities. The author omits noticing the three-toed hogs of Texas, alluded to by Professor Cope; also, does not mention that muscles corresponding to the interossei and supernumery ossicles, as well as a well developed *trapezium* (erroneously termed pisiforne by Percival,) are frequently fouud in heavy-built horses. We shall in a future number of the ARCHIVES, discuss this subject at length; it is one of great interest to the morphologist.

*The Veterinarian*, for November, contains the highly able, and suggestive Introductory Address, held before the Class of the Royal Veterinary College, by Great Britain's most eminent helminthologist, Professor J. S. Cobbold, M.D., F.R.S. It dwells on the important service that helminthology has done to veterinary pathology. Strong confirmation of his position will be found incidentally referred to in our Review Department.

*The forthcoming numbers of the Archive will contain* in the original department: 1. Contributions to the anatomy of the horse's foot; 2. a study of so-called corn and affections of the hoof; 3. an article on the anatomy of the hock and knee region; 4. one on the diseases of the horse's teeth; 5. reports of operations performed in various veterinary infirmaries; 6. notice of new parasites derived from marine animals, and the usual amount of matter in the other departments.

---

A NEW MATERIAL FOR HORSE-BEDDING.—A trial has been made in several infirmaries, and large stables for horses, of the new material for covering the floors of stalls, devised by D. C. Newell & Sons. It consists of *ground wood shavings.* The parties who have used it speak in terms of unqualified praise, and as the Ex-President of the County Medical Society, now Chairman of the Committee on Hygiene, reports it as an excellent corrective of the ammoniacal and other smells of the stable. It is economical, easily handled, and *clean.* In views of these advantages, those who will give it a trial will endorse the award, to Messrs. Newell & Sons, of the Medal of Excellence by the American Institute at the last annual Fair.

---

IN THE MENAGERIE AT CENTRAL PARK.—Director Conklin called our attention to a panther (*Felis concolor*) which was completely blind. The fine sense of touch possessed by virtue of its snout-bristles, enabled it however to move about rapidly and lithely in its cage, without accident. As far as we could judge, it appeared to be *glaucomatous* on both eyes, still there were no opacities of the lense or vitreous humor, and the reflection (green) was evidently from the choroid Tapetum, the pupils were at maximal dilatation.

*The same zoological observer* as we learn from the last annual report of the Zoological Department, of the Central Park. records the rare instance of a pair of swans breeding twice within a year.

